# Search for primate T-lymphotropic virus type 4 in highly exposed human populations and gorillas from Central Africa

**DOI:** 10.1186/1742-4690-12-S1-P83

**Published:** 2015-08-28

**Authors:** Léa Richard, Edouard Betsem, Claudia Filippone, Eric Nerrienet, Antoine Gessain

**Affiliations:** 1 Unit of Epidemiology and Physiopathology of Oncogenic Viruses, Institut Pasteur, Paris, France; 2 Centre National de la Recherche Scientifique (CNRS), UMR 3569, Paris, France; 3 Université Paris Diderot, Cellule Pasteur, Paris, France; 4 Faculty of Medicine and Biomedical Sciences, University of Yaounde I, Yaounde, Cameroon; 5 Centre Pasteur du Cameroun, Yaoundé, Cameroon

## 

The primate T Lymphotropic viruses, which include HTLV-1/STLV-1, HTLV-2/STLV-2, HTLV-3/STLV-3 and HTLV-4/STLV-4 constitute a group of related human and simian retroviruses which share some common epidemiological, biological and molecular features. While HTLV-1 and HTLV-2 are relatively widespread, HTLV-3 has only been observed in few African individuals living in close contact with infected non-human primates (NHPs) and HTLV-4 has been identified in only one hunter living in South Cameroon. Recently, a molecular study conducted in Cameroon on 1107 NHPs of 21 different species detected and characterized STLV-4 infection in 6 gorillas. Therefore HTLV-4 could have emerged from a gorilla reservoir. In the context of our current ongoing studies on PTLV in Cameroon, we decided to look for PTLV-4 in a large series of blood DNA originating from persons at risk for such infection (hunters of NHPs), living in the same region than the index case, and also from several gorillas. HTLV WB serology was available for all the persons. We searched for HTLV-4/STLV-4 infection in humans and gorillas using two different nested PCR, one targeting the pol gene, the other one targeting the LTR. These methods were able to detect 1 copy in a background of 250 ng of DNA. We included in the first preliminary study: 1) 228 individuals from Cameroon that had reported a contact (mostly bite) with a NHP (80 of them specifically with a gorilla); 2) 21 gorillas (16 from Cameroon, 2 from Gabon and 3 from European zoos). Out of the 21 gorillas, one originating from Cameroon was found positive for both STLV-4 pol and LTR PCR. The screening of the human samples is still in progress and will be presented.

